# Internet addiction and its relationship with food choice motives and the risk of eating disorders among young adults in Malaysia

**DOI:** 10.1038/s41598-024-56050-0

**Published:** 2024-03-07

**Authors:** Muhammad Haziq Mohammad Johari, Seok Tyug Tan

**Affiliations:** 1 School of Graduate Studies, Management and Science University, University Drive, Off Persiaran Olahraga, Seksyen 13, 40100 Shah Alam, Selangor Malaysia; 2 Department of Healthcare Professional, Faculty of Health and Life Sciences, Management and Science University, University Drive, Off Persiaran Olahraga, Seksyen 13, 40100 Shah Alam, Selangor Malaysia; 3 Jeffrey Cheah School of Medicine and Health Sciences, Monash University Malaysia, Jalan Lagoon Selatan, 47500 Bandar Sunway, Selangor Malaysia

**Keywords:** Internet addiction, Food choice motives, Disordered eating, Young adults, COVID-19, Psychology, Diseases, Health care, Medical research, Risk factors

## Abstract

The COVID-19 lockdown measures have dramatically altered the daily routines of young adults. Therefore, this study aims to elucidate the relationships between internet addiction, food choice motives and the risk of eating disorders in young adults during the transition to the endemic phase of COVID-19. The Internet Addiction Test was utilised to evaluate the presence and severity of internet addiction among young adults. The Food Choice Questionnaire was employed to assess the food choice motives of young adults, while the risk of eating disorders was determined using the Eating Attitude Test-26. The relationships between internet addiction, food choice motives, and the risk of eating disorders were assessed using model 4 of the PROCESS macro for SPSS. The findings indicated that 29.0% of young adults experienced moderate-to-severe internet addiction, whereas 32.6% were at risk of eating disorders. Young adults were highly valued for the price, sensory appeal, and mood when deciding food choices. The relationship between internet addiction and the risk of eating disorders was partially mediated by convenience (*b* = − 0.211, SE = 0.140, − 0.548 to − 0.016) and familiarity (*b* = 0.219, SE = 0.122, 0.019 to 0.494). A significant direct effect was also observed between internet addiction and the risk of eating disorders (B = 0.793, *p* = 0.017). There is an urgent need to implement intervention strategies aimed at reducing problematic internet use, promoting healthier food choices, and fostering healthy eating habits among young adults.

## Introduction

The COVID-19 lockdown measures have dramatically altered the daily routines, dietary patterns, food purchasing behaviour, and psychological health of human beings^1–3^. During the early COVID-19 outbreak, numerous countries enforced stay-at-home orders to break the transmission chain of the virus. The stringent lockdown measures made studying and working remotely from home the new normal throughout the unprecedented pandemic. One of the adverse effects following the study-at-home and work-from-home orders is that people spend more time on the internet than they did before the pandemic. A recent study by Mary et al. (2022)^4^ revealed that Malaysian young adults spent twice as much time online during the Movement Control Order than before the pandemic. In addition, the proportion of Malaysians participating in social networks has slightly increased from 97.1% in 2019 (pre-pandemic) to 99.2% in 2022 (2 years after the pandemic outbreak in Malaysia). Likewise, the proportion of Malaysians who surfed online for goods or services has also surged from 83.5% (in 2019) to 92.5% (in 2022)^5,6^.

A higher reliance on the internet for various activities and information gathering during the pandemic might increase the likelihood of internet addiction, changing food choice motives and engaging in disordered eating behaviour^7–9^. The mandatory movement restriction orders have spurred the growth of electronic commerce (e-commerce) in Malaysia. According to the Usage of ICT and E-commerce by Establishments (ICTEC) (2020), the total income for e-commerce transactions soared tremendously by 32.7%, from RM 675.4 billion in 2019 (pre-pandemic) to RM 896.4 billion in 2020 (during the pandemic)^10^. The accelerated growth in e-commerce could be attributed to people transitioning from traditional physical store visits to online shopping for necessities during the lockdown. The change in purchasing behaviour may impact food choice motives, as individuals can access a broader range of food options online and make choices based on convenience, availability, and health considerations^8^. Excessive social media engagement can also compromise the ideal body image, thus increasing the likelihood of engaging in disordered eating behaviour^11^.

In Bangladesh, a recent study by Banna et al.^12^ revealed that moderate-to-severe internet addiction facilitates convenient access to unhealthy foods, thereby increasing the risk of university students engaging in disordered eating behaviour such as overeating and binge eating. Even though Malaysia began transitioning into the endemic phase of COVID-19 starting April 1st, 2022^13^, it remains uncertain how internet usage, food choice motives, and the risk of eating disorders are interrelated in the aftermath of the pandemic. Therefore, this study aims to elucidate the relationships between internet addiction, food choice motives and the risk of eating disorders among young adults during the transition to the endemic phase of COVID-19 in Malaysia. Young adults are defined as individuals between the ages of 18 and 30 years as outlined in the newly enacted Youth Societies and Youth Development (Amendment) Act 2019 of Malaysia^14^.

## Methodology

### Study design and population

This anonymous cross-sectional study was conducted from August 25th, 2022, to April 26th, 2023 (during the transition to the endemic phase of COVID-19). A combination of convenience, snowball and purposive sampling approaches was adopted to recruit Malaysian young adults aged 18–30 who have access to the internet, are literate in English, physically healthy and free from clinically diagnosed eating disorders and internet addiction into this study. Non-Malaysian, young adults who do not have access to the internet, are illiterate in English, are not physically healthy, and have been clinically diagnosed with eating disorders or internet addiction by a clinician were excluded from this study. All survey questions are presented in English and hosted on Google Forms. The survey link generated from Google Forms was shared with potential respondents through various social media platforms, including Instagram, TikTok, Facebook, WhatsApp, Telegram and WeChat. It was shared either directly with eligible potential respondents or through groups that specifically involved young adults on those previously mentioned platforms. Young adults who intended to participate in the survey were required to provide informed consent and an affirmative response that they met the inclusion criteria before answering the first survey question.

The sample size was estimated using the Raosoft sample size calculator (http://www.raosoft.com/samplesize.html). Considering that Malaysia had an estimated population of 11.3 million young adults in 2022^15^ and that 20.4% of young adults are at risk of eating disorders^16^, this study would require at least 250 respondents at a 5% margin of error and a 95% confidence level. A total of 337 young adults responded to this study; however, the current findings were computed based on the responses from 331 Malaysian young adults, following the exclusion of duplicate responses and non-Malaysians.

### Socio-demographic characteristics

Young adults were required to self-report their gender, age, ethnicity, marital status, educational attainment, and monthly income (RM).

### Internet addiction

The presence and severity of internet addiction in young adults were assessed using the unmodified 20-item Internet Addiction Test (IAT)^17^. All items are on a 6-point Likert scale ranging from 0 (not applicable) to 5 (always), making a total possible score between 0 and 100. The raw scores of IAT were further categorised into four groups: normal (0–30 points), mild (31–49 points), moderate (50–79 points) and severe (80–100 points). The reliability of IAT is excellent, with a Cronbach’s alpha of 0.919. The item-total correlations range from 0.278 to 0.724, which are above the Pearson correlation critical value of 0.113. Overall, this indicates that the item-total correlations are statistically significant, suggesting that all items in the IAT are valid.

### Food choice motives

The food choice motives of young adults were determined using a validated and unmodified 36-item Food Choice Questionnaire (FCQ)^18^. These 36 items were further classified into nine food choice motive factors: price (3-item), sensory appeal (4-item), mood (6-item), health (6-item), convenience (5-item), weight control (3-item), natural content (3-item), familiarity (3-item), and ethical concern (3-item). All items were evaluated using a 5-point Likert scale ranging from 1 (not very important) to 5 (very important). The mean score was tabulated by dividing the total score by the number of items in a factor. The reliability of FCQ is excellent, with a Cronbach’s alpha of 0.940. The item-total correlations range from 0.405 to 0.670, which are above the Pearson correlation critical value of 0.113. Overall, this indicates that the item-total correlations are statistically significant, suggesting that all items in the FCQ are valid.

### The risk of eating disorders

The unmodified 26-item Eating Attitude Test (EAT-26) was used to assess the risk of eating disorders (also referred to as disordered eating) among young adults^19^. All items were rated on a 6-point Likert scale (always/usually/often/sometimes/rarely/never). Items 1–25 were scored as follows: “always” = 3, “usually” = 2, “often” = 1, and “sometimes/rarely/never” = 0. On the contrary, item 26 was scored in a reversed manner, wherein “always/usually/often” = 0, “sometimes” = 1, “rarely” = 2, and “never” = 3. The total possible score for EAT-26 ranges between 0–78. Young adults with a score of more than or equal to 20 (≥ 20) were considered to be at risk for eating disorders, whereas a score of below 20 (< 20) implied no risk for eating disorders. The reliability of EAT-26 is excellent, with a Cronbach’s alpha of 0.874. The item-total correlations range from 0.132 to 0.699, which are above the Pearson correlation critical value of 0.113. Overall, this indicates that the item-total correlations are statistically significant, suggesting that all items in the EAT-26 are valid.

### Statistical analysis

**Figure 1:**
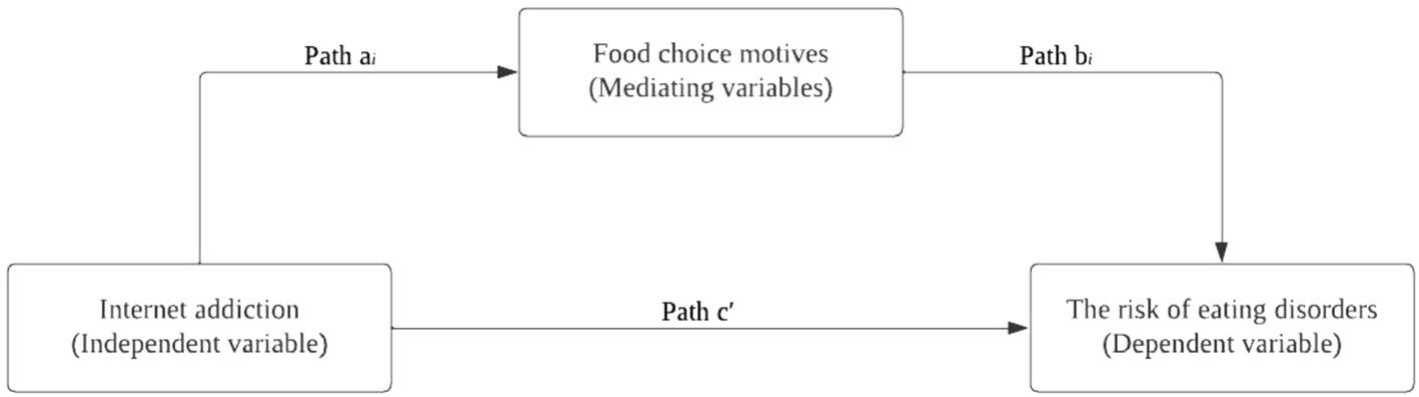
Statistical diagram illustrating the path analysis of the relationships between internet addiction, food choice motives, and disordered eating.

Data were analysed using IBM SPSS statistics version 29.0 (IBM Corp., Armonk, NY, USA). Descriptive statistics, including frequency, percentage, mean, and standard deviation, were used to describe the variables where appropriate. The independent samples t-test was employed to determine the mean difference in food choice motives among young adults based on the severity of internet addiction and the risk of eating disorders. The relationships between internet addiction, food choice motives, and the risk of eating disorders were assessed using model 4 of the PROCESS macro for SPSS^20^. The statistical diagram for path analysis is presented in Fig. 1. Food choice motives were treated as the mediators in the relationship between internet addiction (independent variable) and the risk of eating disorders (dependent variable). A *p-*value of less than 0.05 (*p* < 0.05) was considered statistically significant.

### Ethical approval and consent to participate

Ethical approval was granted by the Research Ethics Committee of Management and Science University with the reference number EA-L1-01-FHLS-2022-11-0008.

### Informed consent

Informed consent was obtained from all respondents prior to data collection.

### Transparency declaration

The corresponding author affirms that this manuscript is an honest, accurate, and transparent account of the reported study. The reporting of this work is compliant with STROBE guidelines. The lead author affirms that no important aspects of the study have been omitted and that any discrepancies from the study as planned have been explained.

## Results

The socio-demographic characteristics of young adults are presented in Table 1. Out of the 331 young adults who participated in this study, the majority were females (n = 186, 56.2%), aged between 25 and 30 (n = 236, 71.3%), Malay (n = 189, 57.1%), with a single marital status (n = 237, 71.6%), tertiary educated (n = 294, 88.8%) and earned less than RM 5000 in a month (n = 291, 87.9%).

**Table 1:** Socio-demographic characteristics of young adults.

Characteristics	Frequency, n (%)	Mean ± standard deviation
Gender		
Male	145 (43.8)	–
Female	186 (56.2)	
Age (years old)		
15–19	15 (4.5)	25.44 ± 3.04
20–24	80 (24.2)	
25–30	236 (71.3)	
Ethnicity		
Malay	189 (57.1)	–
Chinese	59 (17.8)	
Indian	78 (23.6)	
Others (Mixed race or *Bumiputra* of Sabah and Sarawak)	5 (1.5)	
Marital status		
Single	237 (71.6)	–
Married	89 (26.9)	
Divorced/Widowed	5 (1.5)	
Educational attainment		
Secondary	37 (11.2)	–
Tertiary	294 (88.8)	
Monthly income (RM)		
< RM 4999	291 (87.9)	2764.56 ± 2011.74
≥ RM 5000	40 (12.1)	

**Figure 2:**
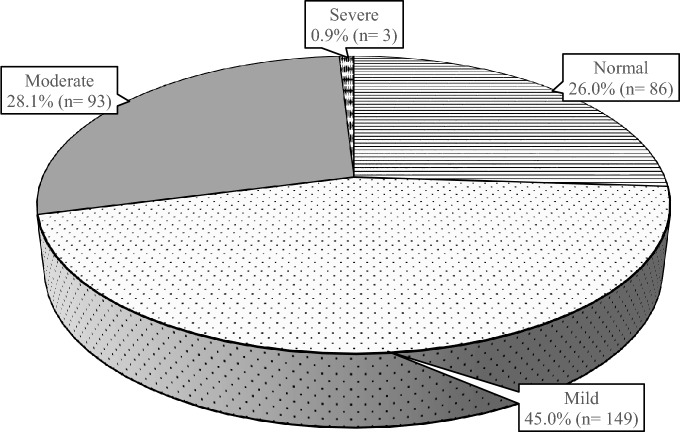
The prevalence of internet addiction among young adults.

**Figure 3:**
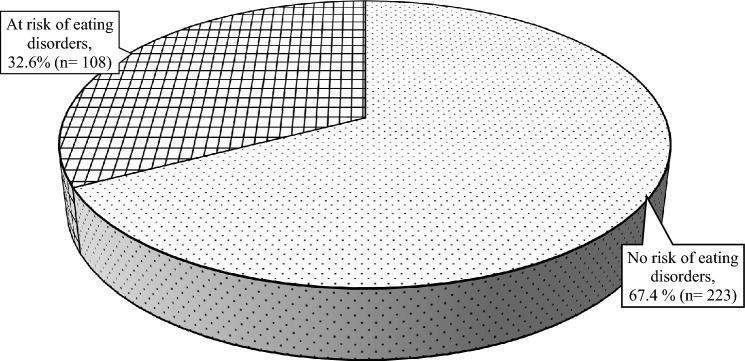
The risk of eating disorders among young adults.

Figure 2 depicts the prevalence of internet addiction among young adults. Emerging findings reveal that 26.0% (n = 86) did not show signs of internet addiction, while nearly half of the young adults had mild internet addiction (n = 149, 45.0%). Slightly more than one-fourth of young adults experienced moderate internet addiction (n = 93, 28.1%), whereas only 0.9% (n = 3) were classified as severe internet addicts. The prevalence of internet addiction was further categorised into two main groups: normal-to-mild internet addiction (n = 235, 71.0%) and moderate-to-severe internet addiction (n = 96, 29.0%) for path analysis. The risk of eating disorders among young adults is illustrated in Fig. 3. The findings indicated that two-thirds of young adults had no risk of eating disorders (n = 223, 67.3%), while one-third (n = 108, 32.6%) were at risk of eating disorders. Of the 96 young adults who were moderately to severely addicted to the internet, 42 (43.8%) of them were also at risk of eating disorders.

Table 2 shows the food choice motives of young adults by the severity of internet addiction and the risk of eating disorders. In general, young adults in the current study were highly valued for the price (4.23 ± 0.70), sensory appeal (4.21 ± 0.60), and mood (4.16 ± 0.60) when deciding food choices. Interestingly, the top three rated food choice motives varied slightly based on the severity of internet addiction and the risk of eating disorders. Young adults in the categories of  normal-to-mild internet addiction and no risk of eating disorders prioritised price, sensory appeal, and mood when deciding food choices. While price and sensory appeal have remained the top two food choice motives of those with moderate-to-severe levels of internet addiction, they were also found to rely heavily on convenience. Conversely, mood, price, and weight control were the utmost concerns among those at risk of eating disorders. The findings of the independent-samples t-test revealed that young adults in the moderate-to-severe internet addiction category attained significantly higher mean scores in price (t = − 2.188, *p* = 0.029), convenience (t = − 2.827, *p* = 0.005), and familiarity (t = − 3.887, *p* < 0.001) compared to those in the normal-to-mild internet addiction category. Compared to young adults with no risk of eating disorders, significantly higher mean scores in mood (t = − 3.126, *p* = 0.002), weight control (t = − 6.144, *p* < 0.001), and familiarity (t = − 2.829, *p* = 0.005) were observed among those at risk of eating disorders.

**Table 2:** Food choice motives of young adults by the severity of internet addiction and the risk of eating disorders.

Food choice motives	Mean ± standard deviation						
	Normal-to-mild internet addiction (n = 235)	Moderate-to-severe internet addiction (n = 96)	t-value (*p*-value)	No risk of eating disorders (n = 223)	At risk of eating disorders (n = 108)	t-value (*p*-value)	Overall (n = 331)
Price	4.18 ± 0.75	4.36 ± 0.56	− 2.188 (0.029)*	4.20 ± 0.66	4.30 ± 0.77	− 1.246 (0.214)	4.23 ± 0.70
Sensory appeal	4.18 ± 0.60	4.27 ± 0.59	− 1.243 (0.216)	4.19 ± 0.53	4.23 ± 0.71	− 0.569 (0.570)	4.21 ± 0.60
Mood	4.13 ± 0.62	4.22 ± 0.56	− 1.190 (0.236)	4.09 ± 0.58	4.31 ± 0.61	− 3.126 (0.002)*	4.16 ± 0.60
Health	4.12 ± 0.69	4.07 ± 0.64	0.570 (0.570)	4.07 ± 0.64	4.17 ± 0.73	− 1.280 (0.202)	4.10 ± 0.67
Convenience	4.04 ± 0.68	4.26 ± 0.61	− 2.827 (0.005)*	4.08 ± 0.60	4.14 ± 0.78	− 0.759 (0.449)	4.10 ± 0.67
Weight control	3.88 ± 0.80	3.99 ± 0.74	− 1.243 (0.215)	3.74 ± 0.75	4.27 ± 0.74	− 6.144 (< 0.001)*	3.91 ± 0.78
Natural content	3.90 ± 0.78	3.91 ± 0.79	− 0.065 (0.948)	3.85 ± 0.72	4.03 ± 0.88	− 1.820 (0.070)	3.91 ± 0.78
Familiarity	3.61 ± 0.74	3.94 ± 0.64	− 3.887 (< 0.001)*	3.63 ± 0.69	3.87 ± 0.77	− 2.829 (0.005)*	3.71 ± 0.73
Ethical concern	3.34 ± 0.80	3.44 ± 0.96	− 0.920 (0.358)	3.31 ± 0.80	3.49 ± 0.92	− 1.758 (0.080)	3.37 ± 0.85

*Significant values are in bold and were considered at *p* < 0.05.

Table 3 demonstrates the path analysis of the relationships between internet addiction, food choice motives, and the risk of eating disorders. Moderate-to-severe internet addiction was positively associated with convenience (B = 0.257, *p* = 0.002) and familiarity (B = 0.352, *p* < 0.001) (Path a_*i*_). In reference to Path b_*i*_, sensory appeal (B = − 0.706, *p* = 0.043), health (B = − 1.257, *p* = 0.006), and convenience (B = − 0.820, *p* = 0.018) were negatively associated with the risk of eating disorders. Conversely, mood (B = 0.881, *p* = 0.033), weight control (B = 1.853, *p* < 0.001), and familiarity (B = 0.621, *p* = 0.018) were positively associated with the risk of eating disorders. There was also a significant direct effect between internet addiction and the risk of eating disorders (B = 0.793, *p* = 0.017). The findings of mediation analyses revealed that the relationship between internet addiction and the risk of eating disorders was partially mediated by convenience (*b* = − 0.211, SE = 0.140, − 0.548 to − 0.016) and familiarity (*b* = 0.219, SE = 0.122, 0.019 to 0.494) (Table 4).

**Table 3:** The path analysis of the relationships between internet addiction, food choice motives and the risk of eating disorders.

Path a_*i*_	B (*p*-value)	Path b_*i*_	B (*p*-value)	Path c′	B (*p*-value)
IA → Price	0.165 (0.067)	Price → DE	0.100 (0.711)	IA → DE	0.793 (0.017)*
IA → Sensory appeal	0.117 (0.126)	Sensory appeal → DE	− 0.706 (0.043)*		
IA → Mood	0.126 (0.100)	Mood → DE	0.881 (0.033)*		
IA → Health	**− **0.053 (0.536)	Health → DE	− 1.257 (0.006)*		
IA → Convenience	0.257 (0.002)*	Convenience → DE	− 0.820 (0.018)*		
IA → Weight control	0.109 (0.271)	Weight control → DE	1.853 (< 0.001)*		
IA → Natural content	0.011 (0.912)	Natural content → DE	0.067 (0.834)		
IA → Familiarity	0.352 (< 0.001)*	Familiarity → DE	0.621 (0.018)*		
IA → Ethical concern	0.170 (0.115)	Ethical concern → DE	− 0.021 (0.917)		

*Significant values are in bold and were considered at *p* < 0.05.

1. IA = Internet addiction; dichotomised into two groups: normal-to-mild internet addiction = 0 and moderate-to-severe internet addiction = 1.

2. DE = Disordered eating (also known as the risk of eating disorders); dichotomised into two groups: no risk of eating disorders = 0 and at risk of eating disorders = 1.

3. All analyses were adjusted with the socio-demographic characteristics of young adults.

**Table 4:** Summary of the mediation analysis.

Indirect effect	*b* (SE)	95% confidence level	Conclusion
IA → price → DE	0.017 (0.054)	− 0.082 to 0.142	No mediation
IA → sensory appeal → DE	− 0.082 (0.087)	− 0.300 to 0.040	No mediation
IA → mood → DE	0.111 (0.103)	− 0.039 to 0.367	No mediation
IA → health → DE	0.067 (0.131)	− 0.167 to 0.372	No mediation
IA → convenience → DE	− 0.211 (0.140)	− 0.548 to − 0.016	Competitive partial mediation
IA → weight control → DE	0.202 (0.211)	− 0.197 to 0.638	No mediation
IA → natural content → DE	0.001 (0.039)	− 0.078 to 0.092	No mediation
IA → familiarity → DE	0.219 (0.122)	0.019 to 0.494	Complementary partial mediation
IA → ethical concern → DE	− 0.004 (0.052)	− 0.128 to 0.096	No mediation

### Discussion

The prevalence of moderate-to-severe internet addiction among young adults in Malaysia was formerly reported as 28.1% (moderate internet addiction = 26.1% and severe internet addiction = 2.0%) prior to the COVID-19 pandemic^21^. The findings in this study are generally in line with that previously reported by Masuri et al. (2019)^21^, in which 29.0% of the young adults were moderately or severely addicted to the internet (moderate internet addiction = 28.1% and severe internet addiction = 0.9%). Although reliance on the internet is projected to increase following the global pandemic outbreak^7,22,23^, emerging findings suggest that the prevalence of moderate-to-severe internet addiction during the transition to the endemic phase of COVID-19 was remarkably resilient compared to that of the pre-pandemic level. One of the plausible justifications is that Malaysians are anticipating a return to normalcy through the resumption of daily routines as they had before the outbreak, with the lifting of the Movement Control Order (MCO)^24^.

Three local studies conducted before the pandemic outbreak delineated that 13.9% to 20.4% of university students, whose age typically falls between 18 and 25 years old, were at risk of eating disorders^16,25,26^. The proportion of young adults at risk of eating disorders in this study was 32.6%, which is 1.6–2.3-fold higher than the reported prevalence in university students. Increased psychological distress (such as stress, depression, anxiety, and fear of COVID-19 infection), changes in daily routine and sleep patterns, food insecurity, and increased exposure to weight loss-related content on social media platforms during the pandemic lockdown could be among the factors associated with the surge in the risk of eating disorders^27–30^.

A recent study by Tan et al.^8^ indicated that the COVID-19 lockdown measures have led to a slight change in the food choice motives of Malaysian young adults. Before the emergence of COVID-19, young adults decided their food choices by considering price, sensory appeal, religion, and convenience. During the unprecedented pandemic, mood surpassed religion as one of the top four-rated food choice motives in young adults. The top three-rated food choice motives among young adults in the normal-to-mild internet addiction (price ≈ sensory appeal > mood), moderate-to-severe internet addiction (price > sensory appeal > convenience), and no risk of eating disorders (price > sensory appeal > mood) categories were identical to those previously reported during the pandemic. In addition to price, those at risk of eating disorders were also highly valued for mood and weight control when making food choices. Price was the primary concern of all young adults in this study, irrespective of the severity of internet addiction and the risk of eating disorders. According to the Salaries & Wages Survey Report released in 2021, the median monthly salaries of young adults stood at RM 1468 (15–24 years old) and RM 2001 (25–34 years old) nationally^31^. Young adults with low earnings may have limited purchasing power and be more sensitive to price fluctuations^32^. In reference to those previously mentioned, the rising global food prices after the pandemic lockdown may trigger young adults to consume small portions or engage in disordered eating behaviour such as meal skipping^33–35^.

The findings from the independent samples t-test demonstrated that young adults who are moderately to severely addicted to the internet were more sensitive to food prices, opted for a convenient way to acquire food, and were more likely to consume familiar food than their counterparts. Coincidentally, moderate-to-severe internet addiction was also positively associated with convenience and familiarity after adjusting the socio-demographic variations in young adults. Emerging findings are consistent with literature showing that internet addiction leads to poor dietary habits. These habits are often characterised by a higher preference for convenience foods such as fast foods or junk foods that only require minimal preparation time^36–38^. In China, Zhang et al.^39^ demonstrated that internet addiction contributes to picky eating behaviour among middle school students aged 11–20. A similar pattern of findings was also observed in the current study, wherein young adults with moderate-to-severe internet addiction had limited food choices and a restricted range of preferred foods.

The current study also revealed that young adults at risk of eating disorders attained significantly higher mean scores in mood, weight control and familiarity than their counterparts. Path analysis further confirmed these trends, showing that food choice motives mentioned earlier were positively associated with the risk of eating disorders after adjusting the socio-demographic variations in young adults. In general, emerging findings support the notion that emotion dysregulation, a stronger desire to lose weight, and a higher preference for familiar food are among the factors driving the incidence of disordered eating in young adults^40–42^. It is also worth mentioning that young adults who took sensory appeal, health, and convenience into consideration when making food decisions had a lower risk of developing eating disorders (Table 3). These findings translated to the fact that young adults in this study did not succumb to temptation due to good taste, appealing texture, attractive appearance, and pleasant smell^43^. A lower risk of developing eating disorders was observed among young adults prioritising health because they are more likely to adopt a healthy and balanced diet^44^. Although it has been previously reported that overconsumption of convenience foods such as energy-dense ultra-processed foods contributes to disordered eating^45^, the current finding did not observe such a trend. One of the plausible justifications is that young adults in this study had a stronger preference for healthy convenience foods over unhealthy convenience foods^45^.

The positive direct effect between internet addiction and the risk of eating disorders indicated that young adults with moderate-to-severe internet addiction are more susceptible to disordered eating. Likewise, a meta-analysis by Ioannidis et al.^46^ also stated that problematic internet usage is positively correlated with the general psychopathology of eating disorders, including disordered eating. The unrealistic and distorted body ideals hosted online may prompt individuals with internet addiction to practise restrictive eating, obsessively counting calories or engage in excessive exercise^9,47,48^. In addition, the relationship between internet addiction and the risk of eating disorders was partially mediated by convenience (competitive partial mediation) and familiarity (complementary partial mediation). The choice of convenience foods plays a crucial role in determining the relationship between internet addiction and the risk of eating disorders. Choosing healthy convenience foods may potentially reduce the risk of eating disorders, even among young adults with moderate-to-severe internet addiction. On the other hand, moderate-to-severe internet addiction might heighten the risk of eating disorders, primarily because young adults limit their food choices to familiar options.

Several limitations need to be highlighted in this study. First, since this study adopted a cross-sectional design, it cannot establish a cause-and-effect relationship between internet addiction and disordered eating. Second, this study is overly represented by young adults aged 25–30, those with single marital status, and those who earn less than RM 5000 per month. Therefore, the findings in the current study may not be generalisable to all young adults in Malaysia. Third, only three young adults (0.9%) were severely addicted to the internet in this study, which may potentially affect the accuracy of the findings. Fourth, this study did not consider the food intake of young adults when justifying the relationships between internet addiction, food choice motives, and the risk of eating disorders. To address these limitations, further investigation may adopt longitudinal or qualitative approaches, recruiting more young adults in the younger age group (below 24 years old) as well as a larger number of young adults with severe internet addiction and exploring the dietary patterns of young adults to gain better insights into the variables under study. Despite those previously mentioned, this study is the first to investigate the relationships between internet addiction, food choice motives, and the risk of eating disorders among young adults in the aftermath of the COVID-19 pandemic.

## Conclusion

Emerging findings indicate that 29.0% of young adults experienced moderate-to-severe internet addiction, while 32.6% were at risk of eating disorders in the aftermath of the COVID-19 pandemic. Internet addiction was also found to be positively associated with the risk of eating disorders among young adults in Malaysia. There is an urgent need for policymakers to implement intervention strategies aimed at reducing problematic internet use, promoting healthier food choices, and fostering healthy eating habits among young adults.

## Data Availability

The data that support the findings of this study are available from the corresponding author upon reasonable request.
